# Mechanistic Reappraisal of Early Stage Photochemistry in the Light-Driven Enzyme Protochlorophyllide Oxidoreductase

**DOI:** 10.1371/journal.pone.0045642

**Published:** 2012-09-26

**Authors:** Derren J. Heyes, Samantha J. O. Hardman, David Mansell, John M. Gardiner, Nigel S. Scrutton

**Affiliations:** Manchester Institute of Biotechnology and Photon Science Institute, University of Manchester, Manchester, United Kingdom; University of Hyderabad, India

## Abstract

The light-driven enzyme protochlorophyllide oxidoreductase (POR) catalyzes the reduction of protochlorophyllide (Pchlide) to chlorophyllide (Chlide). This reaction is a key step in the biosynthesis of chlorophyll. Ultrafast photochemical processes within the Pchlide molecule are required for catalysis and previous studies have suggested that a short-lived excited-state species, known as I675*, is the first catalytic intermediate in the reaction and is essential for capturing excitation energy to drive subsequent hydride and proton transfers. The chemical nature of the I675* excited state species and its role in catalysis are not known. Here, we report time-resolved pump-probe spectroscopy measurements to study the involvement of the I675* intermediate in POR photochemistry. We show that I675* is not unique to the POR-catalyzed photoreduction of Pchlide as it is also formed in the absence of the POR enzyme. The I675* species is only produced in samples that contain both Pchlide substrate and Chlide product and its formation is dependent on the pump excitation wavelength. The rate of formation and the quantum yield is maximized in 50∶50 mixtures of the two pigments (Pchlide and Chlide) and is caused by direct energy transfer between Pchlide and neighboring Chlide molecules, which is inhibited in the polar solvent methanol. Consequently, we have re-evaluated the mechanism for early stage photochemistry in the light-driven reduction of Pchlide and propose that I675* represents an excited state species formed in Pchlide-Chlide dimers, possibly an excimer. Contrary to previous reports, we conclude that this excited state species has no direct mechanistic relevance to the POR-catalyzed reduction of Pchlide.

## Introduction

**Figure 1:**
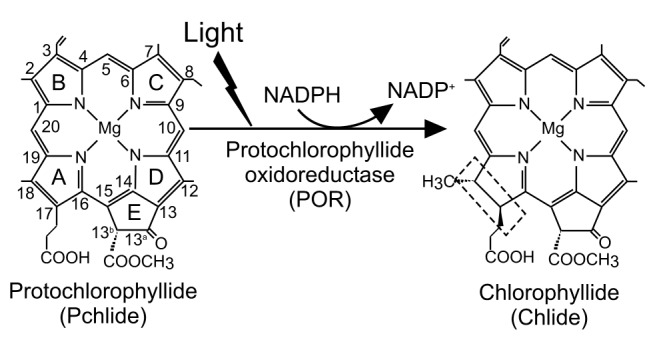
The reaction catalyzed by protochlorophyllide oxidoreductase. The light-driven reduction of the C17–C18 double bond of protochlorophyllide (Pchlide) to form chlorophyllide (Chlide) is catalyzed by protochlorophyllide oxidoreductase (POR) and requires NADPH as a cofactor. The dashed box indicates the double bond that is reduced during the reaction.

The light-driven enzyme protochlorophyllide oxidoreductase (POR) catalyzes the reduction of the C17–C18 double bond of the chlorophyll precursor protochlorophyllide (Pchlide) to form chlorophyllide (Chlide) (Figure 1) [1]–[4]. Biologically, this reaction is a key regulatory step in the chlorophyll biosynthetic pathway and triggers profound changes in plant development, which result in the modification and reorganization of the plastid membranes [1]–[3]. The reaction involves the addition of a hydride and proton at the C17 and C18 positions of Pchlide, respectively. Catalysis is initiated by the absorption of light by the Pchlide substrate [3]. For the majority of enzymes, catalysis is generally limited by diffusional processes (e.g. the binding of substrates and coenzymes), conformational changes in the protein, or product release. Mixing strategies are required to derive detailed mechanistic understanding of such reactions and this is often compromised by the temporal resolution of these methods. As POR is light-activated, the reaction can be initiated from a pre-assembled enzyme-substrate complex using a laser pulse. The chemical steps are then monitored on very fast timescales not generally accessible when using rapid mixing strategies with thermally-activated enzymes. Consequently, POR has become an important model system for studying many aspects of enzyme catalysis [3], [4], including the mechanisms and timescales of proton and hydride transfers [4]–[8], the role of protein dynamics [7], [8], the influence of solvent dynamics on reaction chemistry [9], [10] and comparative analysis of dynamic properties in PORs from different cyanobacterial strains/plants [11].

The POR-catalyzed reaction involves a highly endergonic light-driven hydride transfer from the *pro-S* face of the nicotinamide ring of NADPH to the C17 position of the Pchlide molecule [6], [8], followed by an exergonic thermally-activated proton transfer from a conserved Tyr residue to the C18 position of Pchlide [12] (Figure 1). In POR from *Thermosynechococcus elongatus*, the hydride and proton transfer reactions occur in a sequential mechanism on the microsecond timescale by dynamically coupled nuclear tunneling [8]. Solvent-coupled motions influence the proton transfer reaction but do not affect hydride transfer [9], [10]. Coupling of dynamics to the reaction chemistry may have distinct origins in the evolution of POR enzymes [11]. Motions coupled to light-driven hydride transfer are conserved across all POR enzymes and are apparently localized to the enzyme active site. Motions linked to proton transfer vary in different POR enzymes. Extended ‘dynamic networks’ from solvent to active site are thought to optimize proton transfer in cyanobacterial PORs, but are minimized in plant PORs [11]. Following hydride and proton transfer, a series of ordered product release and coenzyme binding steps are required to complete the catalytic cycle. These binding events are linked to major conformational changes in the enzyme [7], [13].

Chemical steps in the POR catalytic cycle proceed on a relatively slow microsecond timescale [8], but catalysis is also reliant on picosecond excited-state processes associated with Pchlide [4]. Detailed understanding of the Pchlide photochemistry has proved challenging, but over the last few years a number of time-resolved transient spectroscopy studies have identified different short-lived Pchlide* species [4], both in the isolated pigment [14]–[19] and in the ternary enzyme-substrate complex [20]–[22]. Measurements with Pchlide substrate alone have revealed its intrinsic reactivity with multi-exponential excited state dynamics [14]–[19]. Transient spectral changes following excitation of Pchlide have been interpreted with different models. A number of Pchlide excited state species are likely formed within a few nanoseconds, including an intramolecular charge transfer complex [14]–[19] and a triplet state [18], [19]. The excited state dynamics also depend strongly on solvent polarity [14], [15], [19].

To investigate the initial ultrafast steps that lead directly to the chemistry of Pchlide photoreduction, additional transient pump-probe experiments have also been performed on the ternary enzyme-substrate (POR-Pchlide-NADPH) complex [20]–[22]. A putative intermediate with stimulated emission at approximately 675 nm, known as I675*, formed with two time constants of 3 ps and 400 ps [20] and was thought to provide a plausible mechanism for harnessing the light energy to drive the subsequent hydride and proton transfer steps on the microsecond timescale [21], [22]. Formation of the I675* intermediate was found to be strongly dependent on the number of pulses applied to the sample. Consequently, the rate and quantum yield of I675* formation increased significantly after the Pchlide substrate had cycled through the excited state at least once [21]. From these observations the reaction was proposed to involve a two photon mechanism. In this mechanism, the initial photon was suggested to convert POR from an inactive to active configuration; the second photon was then required to initiate catalysis i.e. hydride transfer [21]. However, the origin and exact chemical nature of the I675* excited state species remains elusive, although subsequent time-dependent density function theory calculations [23] and solvent isotope effect studies [22] have suggested it may represent a precursor species in which the Pchlide molecule forms a strongly hydrogen-bonded complex with residues in its direct environment and/or NADPH. Here, we report the use of transient pump-probe absorbance spectroscopy to investigate the formation of the putative I675* intermediate under a range of experimental conditions. In contrast to previous models [21], [22], we show that the rate of formation and quantum yield of I675* is independent of POR and has a strong dependence on the wavelength of the pump source used. These new studies lead us to reappraise the mechanism for early stage photochemistry in the POR catalytic cycle. We conclude that the suggested I675* species is in fact caused by excited state energy transfer between neighboring pigment molecules in a Pchlide:Chlide dimer (possibly an excimer), which is not uniquely formed in the enzyme reaction mechanism. We conclude that I675* is not an essential intermediate in POR-catalyzed reduction of Pchlide.

## Materials and Methods

### Sample Preparation

Recombinant POR from *Thermosynechococcus elongatus* was overexpressed in *Escherichia coli* and purified as described [7]. Pchlide was purified also as described previously [7].

### Synthesis of Chlide

Chlide was synthesized by the insertion of magnesium into pheophorbide *a* (Inochem Ltd) using the general protocol described by Lindsey and Woodford [24]. However, under these conditions magnesium insertion was accompanied by the formation of several allomerization by-products, a known occurrence for chlorophyll and its derivatives due to irreversible modification of *β*-keto ester functionality in the isocyclic ring [25], [26]. Consequently, the magnesium insertion reaction was performed within an anaerobic glovebox (Belle Technology Ltd) under a nitrogen atmosphere (<5 ppm oxygen) using anaerobic triethylamine and rigorously degassed dichloromethane (freeze-thaw method). Under these conditions allomerisation products are nearly completely avoided. Pheophorbide *a* (0.05 g, 0.08 mmol) was dissolved in anhydrous dichloromethane (10 mL), followed by addition of triethylamine (0.24 ml, 1.69 mmol) and MgI_2_ (0.23 g, 0.84 mmol) and the reaction mixture stirred at room temperature. After 30 minutes the reaction mixture was diluted with dichloromethane and washed with water (3×10 mL). The organic layer was dried over Na_2_SO_4_ and removed from the anaerobic glovebox for filtration. The solvent was removed under vacuum to give Chlide *a* as the triethylammonium salt in quantitative yield. Mpt >300°C. ^1^H NMR (400 MHz, pyridine-d_5_): *δ* = 9.95 (s, 1H, H-10), 9.81 (s, 1H, H-5), 8.64 (s, 1H, H-20), 8.34 (dd, *J* 17.8, 11.4 Hz, 1H, H-3a), 6.83 (s, 1H, H-13b), 6.37 (dd, *J* 17.8, 1.4 Hz, 1H, H-3b′); 6.06 (dd, *J* 11.4, 1.4 Hz, 1H, H-3b′′), 4.60 (br q, *J* 6.7 Hz, 1H, H-18), 4.47 (br d, *J* 5.2 Hz, 1H, H-17), 3.81 (s, 3H, OMe), 3.76 (q, *J* 7.6 Hz, 2H, H-8a), 3.71 (s, 3H, H-12a), 3.37 (s, 3H, H-2a), 3.29 (s, 3H, H-7a), 3.00–2.75 (m, 2H, H-17a), 2.70–2.40 (m, 2H, H-17b), 2.63 (q, *J* 7.1 Hz, 6H, (CH_3_C*H*_2_)_3_N^+^H), 1.66 (t, *J* 7.8 Hz, 3H, H-8b), 1.63 (d, *J* 8.0 Hz, 3H, H-18a), 1.03 (t, *J* 7.2 Hz, 9H, (C*H*_3_CH_2_)_3_N^+^H). ^13^C NMR (100 MHz, pyridine-d_5_): *δ* = 190.3 (C-13a), 175. 7 (COO^−^), 171.5 (*C*OMe), 168.1 (C-19), 161.8 (C-14), 156.4 (C-16), 154.3 (C-1), 152.0 (C-6), 148.2 (C-4), 147.9 (C-11), 146.4 (C-9), 144.6 (C-8), 139.2 (C-3), 136.3 (C-2), 134.6 (C-12), 134.5 (C-7), 131.7 (C-13), 131.0 (C-3a), 119.7 (C-3b), 107.6 (C-10), 106.1 (C-15), 100.1 (C-5), 93.3 (C-20), 66.7 (C-13b), 52.3 (OMe), 51.7 (C-17), 49.6 (C-18), 46.3 (CH_3_ *C*H_2_)_3_N^+^H), 31.6 (C-17b), 30.8 (C-17a), 23.4 (C-18a), 19.8 (C-8a), 17.8 (C-8b), 12.8 (C-12a), 12.7 (C-2a), 11.2 (C-7a), 11.0 (*C*H_3_CH_2_)_3_N^+^H). LRMS (ES^−^) m/z: 613 [M–H]^−^; HRMS (FTMS NSI^−^), C_35_H_33_MgN_4_O_5_ [M–H]^−^; calcd.: 613.2307; obsd.: 613.2293.

### Ultrafast Pump-probe Spectroscopy

The laser system used for the transient absorption experiments comprises a Ti:sapphire amplifier (a hybrid Coherent Legend Elite-F-HE) pumped by a Q-switched Nd:YLF laser (Positive Light Evolution-30) and seeded by a Ti:sapphire laser (Spectra-Physics Mai Tai). The amplifier output, which has a wavelength of 800 nm, a 1 kHz repetition rate and ∼120 fs pulse duration is split, with part of the output used to pump a non-collinear optical parametric amplifier (Light Conversion TOPAS-White), which produces the pump beam with a full-width-half-maximum of ca. 10 nm. The pump wavelengths used are described in the text. Another fraction of the amplifier output is used to generate the white light probe pulse for a Helios (Ultrafast Systems LLC) broadband pump-probe transient absorption spectrometer, with an instrument response function of around 170 fs. Samples of the ternary enzyme-substrate complex contained 200 µM Pchlide, 500 µM POR and 2 mM NADPH in activity buffer (50 mM Tris pH 7.5, 100 mM NaCl, 1% Triton X-100, 0.1% 2-mercaptoethanol) and were flowed at a rate of approximately 20 ml/min through a 0.2 mm pathlength quartz cell to ensure that a different area of the sample is excited with each pump laser pulse. For enzyme-denatured measurements, samples containing 20 µM Pchlide, 5 µM POR and 200 µM NADPH in activity buffer were illuminated for different lengths of time, heated to 90°C for 10 mins and centrifuged to remove the precipitated protein. Measurements were performed in a 2 mm pathlength stirred quartz cell. Samples of ‘Pchlide only’ and ‘Chlide only’ contained 20 µM pigment in activity buffer or methanol (as required) in a 2 mm pathlength stirred quartz cell. For all measurements, data points were collected in a random order over a 3 ns time frame.

### Global Analysis

Global analysis was performed using the GLOTARAN software package [27]. The number of lifetimes fitted to each data set was determined by increasing the number of components until the residuals were effectively zero. The longest lifetime in each case was set to over 3 µs, essentially infinity on the 3 ns timescale of the experiments. The time-resolved difference spectra were fitted to two different kinetic models. A sequential, unbranched, unidirectional model yielded evolution associated difference spectra (EADS), which represent the spectral evolution of the decay processes (e.g. the second EADS rises with the first lifetime and decays with the second lifetime). A second parallel model of independently decaying components yielded decay associated difference spectra (DADS) which represent the loss or gain of emission or absorption with a certain lifetime.

### Fluorescence Measurements

Fluorescence emission end excitation spectra were recorded using a FLS920 fluorescence spectrometer (Edinburgh Instruments Ltd.). Samples contained either 20 µM Pchlide, 20 µM Chlide or a 50∶50 mixture of the 2 pigments in a 0.2 mm pathlength cuvette. Emission spectra were measured using an excitation wavelength of 460 nm and excitation spectra were measured using an emission wavelength of 675 nm. Excitation monochromator slit widths were 1 nm and emission monochromator slit widths were 5 nm.

## Results

### I675* Formation in POR-Pchlide-NADPH Complexes

**Figure 2:**
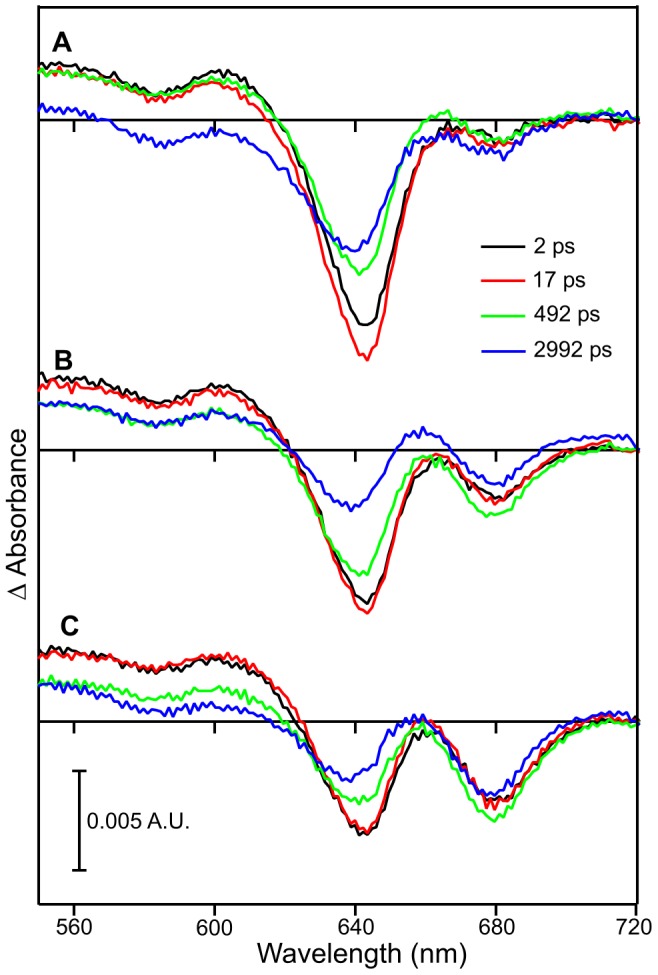
Pump-probe absorption spectroscopy of POR-Pchlide-NADPH samples after photoexcitation with a laser pulse. The laser pulse was centred at ∼475 nm. Transient absorption difference spectra at delay times of 2, 17, 492 and 2992 ps after excitation are shown for the average of scans 1–3 (A), scans 8–10 (B) and scans 18–20 (C).

Initial pump-probe measurements were carried out on POR-Pchlide-NADPH complexes using an excitation wavelength of 475 nm (Figure 2), which yielded similar results to those reported previously [21]. Briefly, time-resolved difference spectra were recorded sequentially so that later scan numbers had been subjected to more laser pulses, resulting in an increased level of the Chlide product in the sample. In the initial scans there is a bleach of the Pchlide ground state absorption at approximately 640 nm, with only a small amount of the negative signal forming over the first few hundred picoseconds at approximately 675 nm, which represents the I675* intermediate (Figure 2A) [21]. However, in the later scans the negative 675 nm signal forms in higher yields within approximately 500 ps (as shown by the green difference in Figure 2C, Figure S1). In addition, there is also direct excitation of the Chlide product that has been formed during the previous scans, which is shown as a ground state bleach at approximately 675 nm immediately after excitation (Figure 2C).

Subsequently, the formation of the I675* species was investigated by exciting POR-Pchlide-NADPH complexes with a pump wavelength of 450 nm, where the Pchlide molecule has a much higher absorbance (Figure S2). Although photoexcitation of Pchlide is more efficient at this wavelength the appearance of the negative signal at 675 nm is much less pronounced (Figure S3 and S4), suggesting that I675* formation is not directly related to Pchlide excitation.

**Figure 3:**
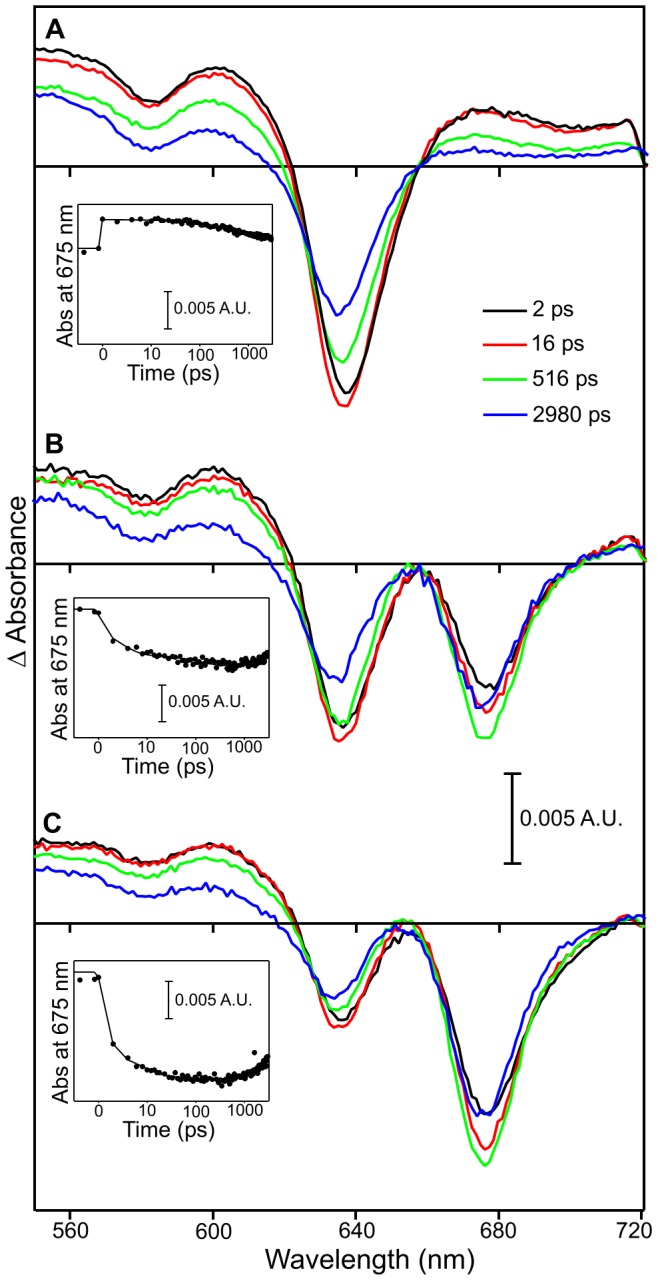
Pump-probe absorption spectroscopy of enzyme-denatured POR-Pchlide-NADPH samples. Spectra are shown after photoexcitation with a laser pulse centred at ∼475 nm. The main panels show transient absorption difference spectra at delay times of 2, 16, 516 and 2980 ps after excitation for POR-Pchlide-NADPH samples that were kept in the dark (A), illuminated for 2 mins (B) and illuminated for 4 mins (C) prior to enzyme denaturation. The insets show the respective kinetic transients at 675 nm (black circles) with a fit of the data to 3 exponentials (solid line).

To investigate the role of the enzyme in I675* formation, POR-Pchlide-NADPH complexes were illuminated for different lengths of time and samples heated to 90°C for 10 mins to denature the POR enzyme. Subsequent pump-probe measurements showed that formation of the I675* intermediate still occurred in these enzyme-denatured samples following excitation at 475 nm (Figure 3). Moreover, the rate of formation and yield of the negative 675 nm signal displays a similar trend to that observed for the native enzyme samples and is dependent on the length of time that samples had been illuminated prior to denaturation (Figure 3). In other words, the level of I675* formation is related to the amount of the Chlide product in the sample (Figure S5).

### Pump-probe Measurements on Pure Pchlide and Chlide Samples

**Figure 4:**
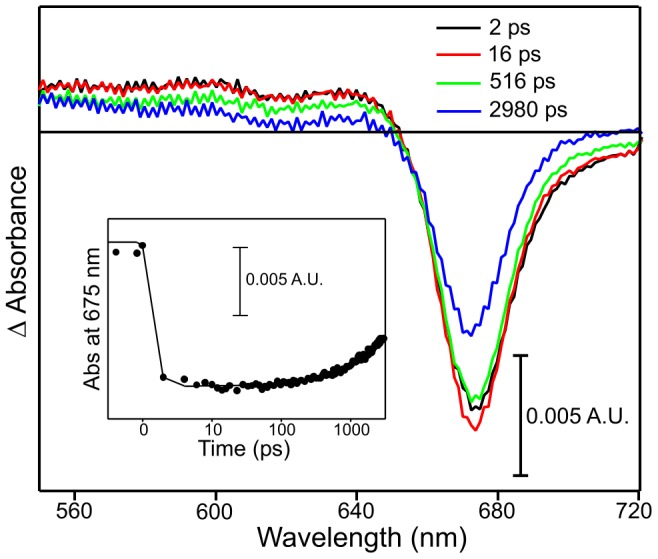
Pump-probe absorption spectroscopy of ‘Chlide only’ sample. Spectra are shown after photoexcitation with a laser pulse centred at ∼475 nm. The main panel shows transient absorption difference spectra at delay times of 2, 16, 516 and 2980 ps after excitation. The inset shows the respective kinetic transients at 675 nm (black circles) with a fit of the data to a single exponential (solid line).

The origin of I675* formation was studied in samples containing only the Chlide product that had been synthesized chemically (see Materials and Methods Section). The time-dependent difference spectra show relatively few spectral features apart from a bleach of the Chlide ground state absorption at approximately 675 nm at time zero (Figure 4). This slowly returns to the ground state over several ns (time constant of 3.2 ns) and there is no further increase in the amplitude of the negative signal at 675 nm within a few hundred ps. Similar spectral changes were also observed when other pump wavelengths were used (Figure S6), confirming that I675* does not form in ‘Chlide only’ samples. Moreover, pump-probe experiments on the isolated Pchlide substrate have also shown that I675* formation does not occur in ‘Pchlide only’ samples upon photoexcitation at any of the pump wavelengths used [14]–[19] (Figure S7).

### Pump-probe Experiments on Mixtures of Pchlide and Chlide

**Figure 5:**
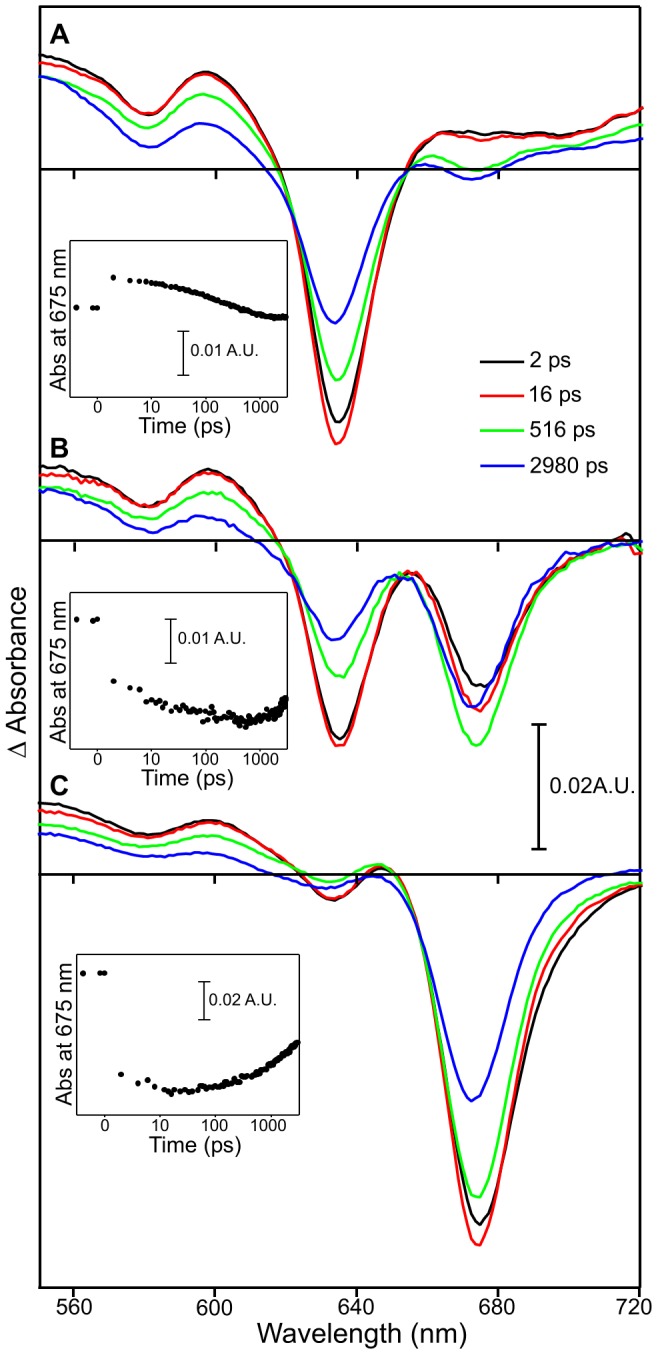
Pump-probe absorption spectroscopy of samples containing a mixture of Pchlide and Chlide. Spectra are shown after photoexcitation with a laser pulse centred at ∼460 nm. The main panels show transient absorption difference spectra at delay times of 2, 16, 516 and 2980 ps after excitation for samples containing a mixture of 90% Pchlide and 10% Chlide (A), 50% Pchlide and 50% Chlide (B) and 10% Pchlide and 90% Chlide (C). The insets show the respective kinetic transients at 675 nm (black circles).

When pump-probe experiments were repeated with mixtures of the isolated Pchlide and Chlide pigments (mimicking the enzyme-containing samples, but importantly with enzyme not present) the I675* intermediate was formed in significant quantities with rate constants similar to those observed previously (Figure 5). Moreover, the yield of I675* formation was found to be dependent on the ratio of Pchlide and Chlide in the sample with a maximal level of the I675* species found in samples containing a 50∶50 mixture of Pchlide to Chlide (Figure 5, Table 1). The yield of I675* formation was found to be strongly dependent on the pump wavelength used (Figure S8). The amount of I675* formed was highest when a pump wavelength of 460 nm was used (Figure S8C), with time constants of 10.8 and 258 ps, but this decreases rapidly on the high energy side (very little signal when exciting at 450 nm) and decreases slowly when longer wavelength are used (*i.e.* a relatively large signal remains upon excitation at 475 nm).

**Table 1:** Amplitudes and time constants for the formation of I675* in samples containing a mixture of Pchlide and Chlide.

Sample	A1 (abs units)	T1 (ps)	A2 (abs units)	T2 (ps)
90% Pchlide, 10% Chlide	0.0016	66.4±38.1	0.0025	468.1±138.0
75% Pchlide, 25% Chlide	0.0035	11.5±3.1	0.0048	279.9±93.2
50% Pchlide, 50% Chlide	0.0048	10.8±3.1	0.0045	258.4±111.2
25% Pchlide, 75% Chlide	0.0049	7.0±3.2	0.0017	214.5±252.0
10% Pchlide, 90% Chlide	0.0052	5.0±3.3	n/a	n/a

**Figure 6:**
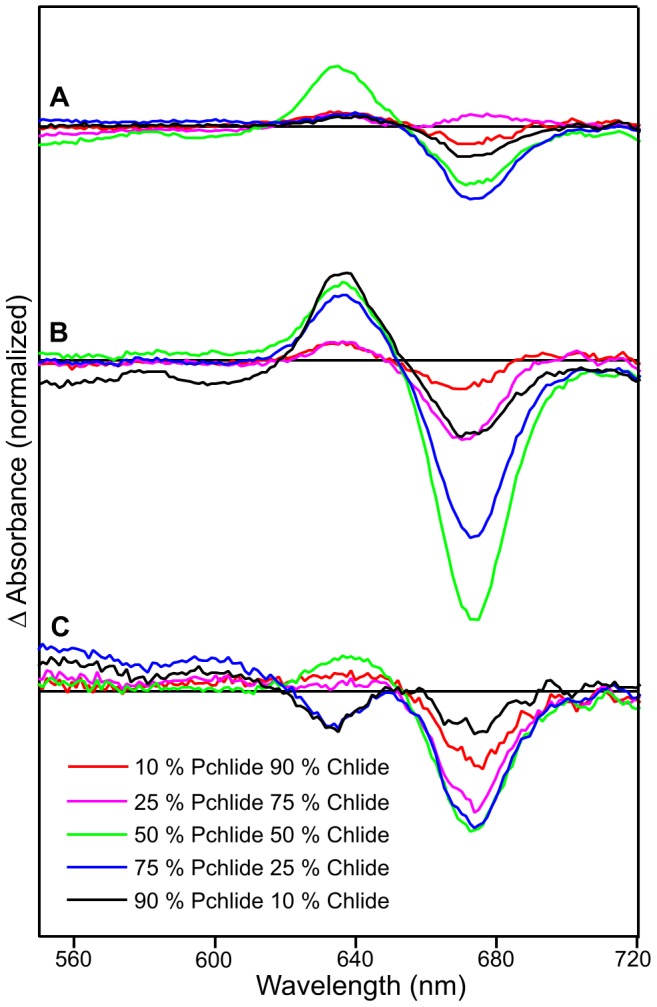
Residuals calculated after subtraction of the sum of the two component data from the time-resolved difference spectra for Pchlide and Chlide mixtures. The residuals calculated at 1 ns after subtraction of the sum of the ‘Pchlide only’ and ‘Chlide only’ data from the actual time-resolved difference spectra for all of the Pchlide and Chlide mixtures after photoexcitation at 450 nm (A), 460 nm (B) and 475 nm (C).

In the absence of any additional excited state species the time-resolved difference spectra for the Pchlide and Chlide mixtures are expected to be a sum of the ‘Pchlide only’ and ‘Chlide only’ data. The time-dependent difference spectra for the Pchlide and Chlide mixtures were analyzed by subtracting the sum of the difference spectra of the isolated Pchlide and Chlide samples. The residuals (*Z_residual_,*) from the subtractions were obtained by using the following equation:
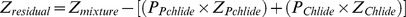
where P is the proportion of Pchlide or Chlide in the sample mixture (*e. g.* 0.9 and 0.1 for a 90∶10% mixture of Pchlide and Chlide). The time-resolved difference spectra were also corrected for pump fluence to allow for a more accurate comparison. The residuals calculated after subtraction of the sum of the two component data from the actual time-resolved difference spectra are shown for all of the Pchlide and Chlide mixtures at each of the different pump wavelengths (Figure 6 and S9). There is clearly an additional short-lived negative signal at approximately 675 nm in some of the Pchlide and Chlide mixtures that isn’t present in the pure pigment samples. Again, the intensity of the negative signal at 675 nm was highest in samples containing a 50∶50 mixture of Pchlide to Chlide (indicated by the green trace in Figure 6) and when a pump wavelength of 460 nm was used (Figure 6B).

### Global Analysis of Pump-probe Data

**Figure 7:**
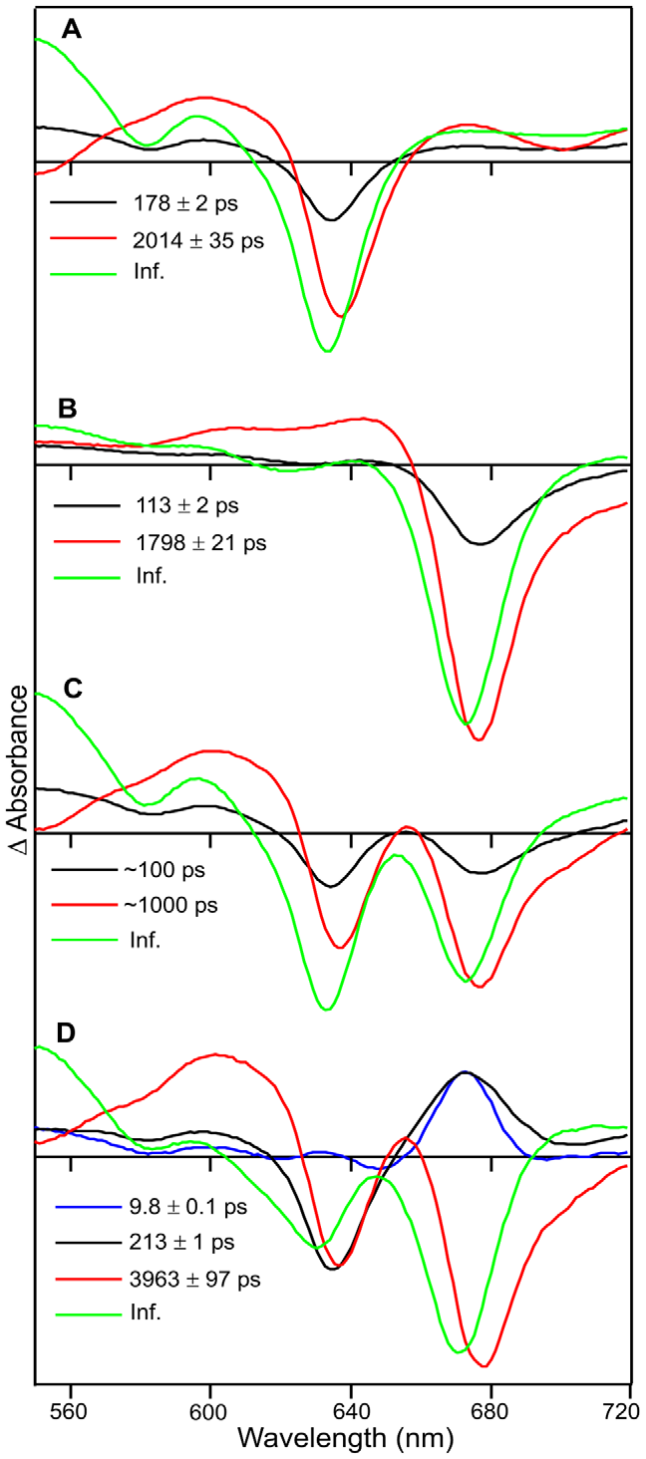
Decay associated difference spectra (DADS) resulting from a global analysis of the pump-probe absorption spectroscopy data. Shown are DADS for Pchlide only (A), Chlide only (B), a sum of the ‘Pchlide only’ and ‘Chlide only’ data (C) and a mixture of 50% Pchlide and 50% Chlide, after photoexcitation at 460 nm. The data were fitted to a parallel model of independently decaying components as described in the Materials and Methods Section.

Global analysis was used to model the time-resolved difference spectra for the isolated Pchlide and Chlide pigments, and a 50∶50 mixture of Pchlide to Chlide after excitation at 450, 460 and 475 nm (Figures 7, S10 and S11). For clarification and to allow a direct comparison between the individual pigments and the Pchlide:Chlide mixture the fitted data for Pchlide only and Chlide only are shown independently and also as a sum of the individual component data. The Pchlide only and Chlide only data could be fitted to 3 exponential functions upon excitation at 450 nm and 460 nm, and 2 exponential functions upon excitation at 475 nm excitation, with the reduced number of components likely to be a result of the low signal intensity at this excitation wavelength. However, an additional component was required to fit the data from the 50∶50 mixture of Pchlide:Chlide. A sequential model yields the EADS shown in figure S10, which clearly show the formation and subsequent decay of the negative peak at 675 nm in the Pchlide:Chlide mixture, but not in the individual pigments. However, to gain further insight into the processes that occur upon photoexcitation the data were also fitted to a parallel model, which yielded the DADS shown in figure 7 and S11. Significant differences can be observed between the DADS derived from a sum of the Pchlide only and Chlide only fits, and the DADS fitted to the 50∶50 mixture, although the component at approximately 1000 ps is similar at all excitation wavelengths. The spectrum with the infinite lifetime in the 50∶50 Pchlide:Chlide mixture shows an increased contribution from the negative signal at 675 nm (*i.e.* Chlide) relative to the ground state bleach of Pchlide, which is indicative of energy transfer from Pchlide to Chlide (Figure 7 and S11). In addition, the shorter lifetime component of the individual pigments show simple ground state bleaching, whereas the shorter lifetime components of the 50∶50 Pchlide:Chlide mixture show a bleach in the region of the Pchlide ground state absorption peak and a positive component in the region of the Chlide ground state absorption peak (Figure 7 and S11). Similar spectral features have also been observed in mixtures of chlorophyll *a* and chlorophyll *b* and are proposed to be characteristic of energy transfer between the two pigments [28]–[30]. Hence, both the short lifetime and the infinite lifetime DADS indicate that wavelength dependent energy transfer occurs between the Pchlide and Chlide moieties.

To confirm that similar energy transfer processes occur in the ternary enzyme-substrate complex global analysis of the pump-probe data for the POR-Pchlide-NADPH samples revealed that comparable time constants to those of the isolated pigments and pigment mixtures could be obtained. A sequential model yielded the EADS in figure S12, which show the appearance of the I675* species in the later scans of the pump-probe experiment. A parallel model yielded the DADS in figure S13, which shows that the DADS for the initial scans of the pump-probe experiments are very similar to those calculated for the Pchlide only samples at all of the excitation wavelengths. However, as the number of scans increase the spectral features associated with energy transfer becomes more intense and in the final scans (18–20) the DADS are very similar to those calculated for the 50∶50 Pchlide:Chlide mixtures.

### Effect of Solvent on I675* Formation

**Figure 8:**
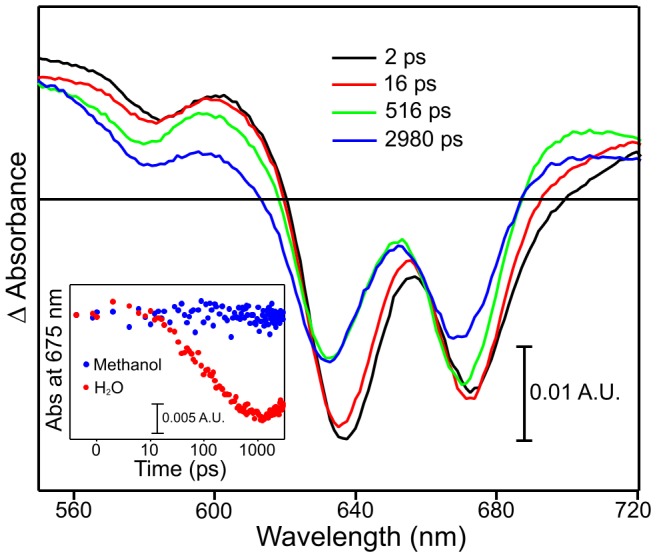
Pump-probe absorption spectroscopy of samples containing a mixture of 50% Pchlide and 50% Chlide in methanol after photoexcitation with a laser pulse centred at ∼460 nm. The main panel show transient absorption difference spectra at delay times of 2, 16, 516 and 2980 ps after excitation. The inset shows the residuals at 675 nm for a mixture of 50% Pchlide and 50% Chlide in methanol and H_2_O after subtraction of the sum of the ‘Pchlide only’ and ‘Chlide only’ data from the actual time-resolved difference spectra.

Previous studies have shown that the excited state dynamics of isolated Pchlide is strongly dependent on the solvent [14], [15], [19]. We therefore also investigated the role of solvent on the rate and yield of I675* formation in samples containing a 50∶50 mixture of Pchlide and Chlide by repeating transient absorption measurements in the polar solvent methanol (Figure 8). The I675* species was not formed upon photoexcitation at 460 nm in methanol as the amplitude of the negative signal at 675 nm does not increase over the first 500 ps. The residuals calculated after subtraction of the sum of the two component data from the actual time-resolved difference spectra for the Pchlide and Chlide mixture in methanol is negligible over all timescales (Figure 8 inset). Hence, the Pchlide and Chlide mixture does not contain any additional excited state species compared to the ‘Pchlide only’ and ‘Chlide only’ samples when methanol is used as solvent.

### Fluorescence Measurements on Pchlide and Chlide Samples

**Figure 9:**
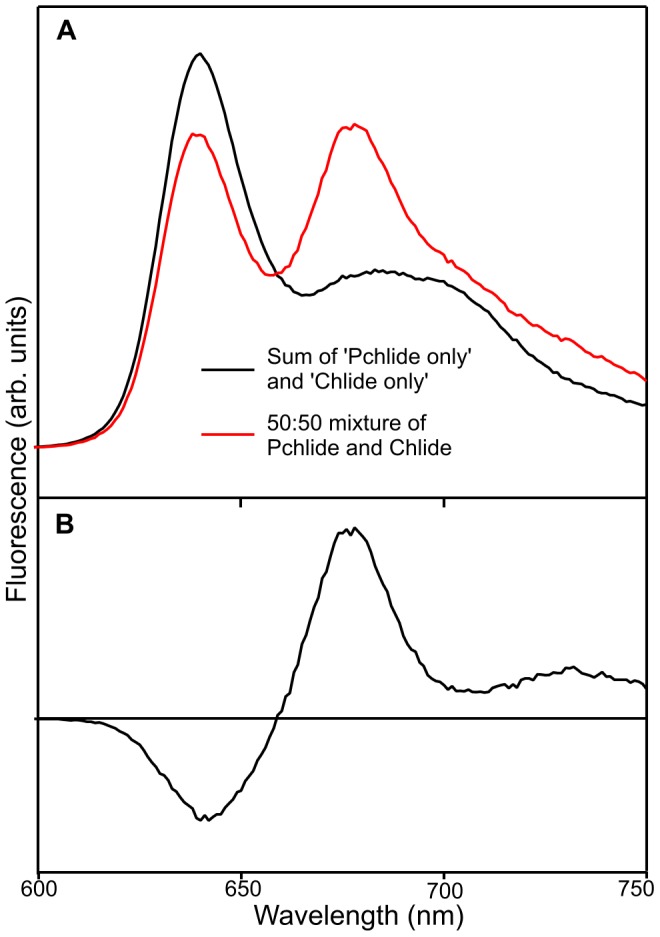
Fluorescence emission spectra of samples containing ‘Pchlide only’, ‘Chlide only’ and a 50∶50 mixture of Pchlide and Chlide. (A) Fluorescence emission spectra of samples containing a mixture of 50% Pchlide and 50% Chlide and the sum of the ‘Pchlide only’ and ‘Chlide only’ spectra in H_2_O after excitation at 460 nm. (B) Difference spectrum for a mixture of 50% Pchlide and 50% Chlide in H_2_O after subtraction of the sum of the ‘Pchlide only’ and ‘Chlide only’ data from the actual fluorescence emission spectra.

Fluorescence emission spectra were measured for samples containing ‘Pchlide only’, ‘Chlide only’ and a 50∶50 mixture of Pchlide and Chlide in both aqueous buffer and methanol (Figure 9 and S14). In aqueous buffer there are additional spectral features in the Pchlide and Chlide mixtures, which are not present in the sum of the ‘Pchlide only’ and ‘Chlide only’ data (Figure 9A). There is energy transfer from Pchlide to Chlide, as shown by a quenching of the Pchlide fluorescence emission band at approximately 635 nm together with a simultaneous increase in the fluorescence emission from Chlide at approximately 675 nm (Figure 9B). However, in aqueous buffer there are no such differences between the ground state absorption spectra of a 50∶50 mixture Pchlide and Chlide and a sum of the ‘Pchlide only’ and ‘Chlide only’ spectra (Figure S15), confirming that any changes are due to excited state processes. In addition, fluorescence excitation spectra, recorded using an emission wavelength of 675 nm, show that additional excitation peaks in the Pchlide and Chlide mixture sample are from the Pchlide molecule and provide further evidence of energy transfer between the pigments (Figure S16). This also explains why the I675* species only forms upon photoexcitation at certain wavelengths. Pchlide has a much higher absorbance at 460 nm than Chlide (Figure S2), resulting in a higher level of energy transfer from Pchlide to Chlide. Conversely, at wavelengths below 450 nm there is more direct excitation of Chlide and I675* formation is lower as result of less energy transfer from Pchlide to Chlide. However, when measurements are repeated in methanol the fluorescence emission spectra for the Pchlide and Chlide mixture is simply a sum of the ‘Pchlide only’ and ‘Chlide only’ data, suggesting a lack of energy transfer between the two pigments in this solvent (Figure S14).

## Discussion

POR is a light-driven enzyme that catalyzes the reduction of the C17–C18 double bond of the chlorophyll precursor molecule, Pchlide [1]–[4]. Consequently, the catalytic mechanism of POR, which involves sequential hydride and proton transfer steps [8], is completely dependent on the excited-state dynamics of Pchlide, which occur on the picosecond timescale [4]. Although the exact mechanism of photochemistry is not fully understood a short-lived intermediate with a negative signal at approximately 675 nm was previously suggested as the first catalytic intermediate in the reaction cycle, which is formed within a few hundred picoseconds [20]–[22]. This putative intermediate, referred to as I675*, was proposed to represent an excited state H-bonded intermediate, which harnesses the majority of the excitation energy to drive the subsequent hydride and proton transfer chemistry [21], [22]. However, the exact chemical nature of I675* remained unclear and more detailed studies were required to understand the mechanism of photochemistry in the enzyme-substrate complex. We set out to characterize the I675* species in detail using time-resolved pump-probe spectroscopy to study the influence of the POR enzyme, pump wavelength and solvent on its formation. Contrary to previous suggestions [21], [22], we find that the I675* species is also formed in the absence of POR. Accordingly, in light of these findings, the mechanism for early stage photochemistry in the light-driven reduction of Pchlide needs to be re-evaluated.

The I675* intermediate has previously only been identified and characterized in transient spectroscopy studies on samples containing POR-Pchlide-NADPH complexes [20]–[22]. From these studies, formation of I675* was proposed as the first step in the POR-catalytic cycle [21], [22]. In turn, formation of I675* was proposed to facilitate subsequent hydride and proton transfer on the microsecond timescale [8]. We have now established that I675* is formed in samples following thermal denaturation of POR and also in Pchlide and Chlide mixtures that contain no POR enzyme. Moreover, I675* formation is maximized in 50∶50 mixtures of Pchlide and Chlide. It is unlikely therefore that the I675* species is an obligate intermediate in the catalytic cycle of POR. We therefore conclude that formation of I675* is not required for subsequent hydride and proton transfer in the POR catalytic cycle. In POR containing samples, the I675* species has only previously been observed following prior excitation of the enzyme-substrate complex [21]. This has the effect of increasing the level of the Chlide product in the sample prior to further data acquisition. Similarly, in the present work we show that in the absence of POR, the formation of I675* only occurs in samples that contain a mixture of Pchlide and Chlide. This supports our conclusion that the I675* species observed in previous studies [20]–[22] is not a catalytic intermediate in the POR-catalyzed reaction; it’s formation however does require the presence of both the Pchlide substrate and the Chlide product in the sample.

In light of these new findings, it is now possible to propose a new hypothesis regarding the origin and chemical nature of the I675* excited state species. We have shown that I675* only forms when Pchlide and Chlide are both present in the sample and that the level of the I675* species is maximized when a 50∶50 ratio of the two pigments is used. This suggests that there are interactions between Pchlide and Chlide during formation of the I675* species. Moreover, the global analyses and fluorescence measurements reveal that there is direct energy transfer between neighboring Pchlide and Chlide molecules in samples containing a mixture of the two pigments. Previous spectroscopic studies on etiolated beans have shown that Pchlide-Chlide dimers, and possibly larger pigment aggregates, can be formed in etioplast membranes [31]–[33]. It has also been shown that these Pchlide-Chlide dimers have an absorption peak at 676 nm [31]. Hence, we propose that I675* is likely to represent energy transfer from excited state Pchlide to neighboring Chlide molecules in Pchlide-Chlide dimers. Although the ground state absorption spectra show no evidence of Pchlide-Chlide dimers (see Figure S15), it is possible that I675* is an excimer (*i. e.* the dimer only forms in the excited state), possibly as a result of an increased hydrogen-bonding network. There is precedent for excited state dimerization in similar molecules as excimers have previously been observed in a Chlide analogue as a result of increased H-bonding between neighboring C17 propionic acid sidechains [34].

A dimerization model is also supported by the results on Pchlide and Chlide mixtures in methanol, where no I675* formation was observed. In addition, fluorescence measurements show that there is no energy transfer between Pchlide and Chlide molecules in the excited state. As a polar solvent methanol has been shown to form multiple intermolecular site-specific coordination and H-bonding interactions with the Pchlide and Chlide pigments in the ground state, which are then strengthened in the excited state [23]. This would inhibit any additional interactions between the Pchlide and Chlide molecules and is likely to prevent excited state dimerization in these measurements. Moreover, this may help to explain why the excited state dynamics of Pchlide itself is strongly dependent on the solvent polarity [14], [15], [19]. In non-polar solvents it would be expected that similar excited state dimerization could occur between neighboring Pchlide molecules, which would then be disrupted in more polar solvents. Hence, this dimerization process may need to be taken into account when interpreting and modeling the excited state dynamics of Pchlide itself [14]–[19].

**Figure 10:**
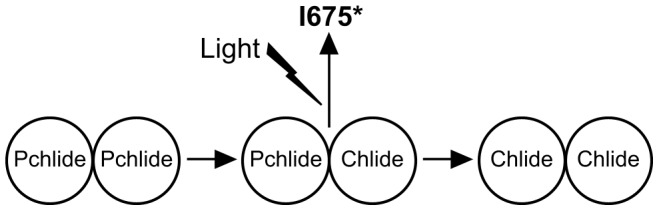
Scheme showing proposed step-wise conversion of Pchlide dimers to Chlide dimers. The I675* species only forms upon excitation of Pchlide-Chlide dimers.

Previous studies on the I675* species have led to the suggestion that the POR-catalyzed reaction is a two photon process [21]. The absorption of an initial photon was proposed to induce a more favorable conformation of the active site, which converts the POR enzyme from an inactive to an active configuration. The formation of I675*, which was thought to be necessary for catalysis, only then occurs upon absorption of a second photon [21]. However, this two photon phenomenon for the POR-catalyzed reaction can now be explained using the putative dimerization model for I675* formation. It is likely that a first photon is required to convert Pchlide-Pchlide dimers into Pchlide-Chlide dimers, which can then be converted into Chlide-Chlide dimers upon absorption of the second photon (Figure 10). As we have now shown that the I675* species is likely to result from energy transfer between pigment molecules in Pchlide-Chlide dimers this would explain why it can only be observed in POR-Pchlide-NADPH complexes upon prior excitation with a laser pulse. Indeed, early spectroscopic measurements on etioplast membranes have also indicated that the photoreduction of Pchlide is a two photon process *in vivo* [31], [32]. It was shown that enzyme-bound dimers of Pchlide were reduced stepwise to dimers of Chlide in two successive light reactions, with the mixed Pchlide-Chlide dimer formed as a stable intermediate species [31], [32].

In conclusion, we have studied the initial excited state processes associated with the light-driven enzyme POR and shown that the previously proposed mechanism of photochemistry [21], [22] needs to be re-evaluated. We have shown that an excited state species, I675*, which was thought to be essential for the subsequent hydride and proton transfer chemistry [21], is not an obligatory catalytic intermediate in Pchlide photoreduction. Instead, we conclude that I675* represents an excited state energy transfer species formed between neighboring pigment molecules in Pchlide-Chlide dimers (possibly excimers) [34]. These findings illustrate the challenges in interpreting complex excited state spectral changes. Our data provide further evidence for the highly reactive nature of these chlorophyll precursor molecules and new mechanistic insight into a natural light-activated enzyme that is crucial to life on Earth.

## Supporting Information

Figure S1 The time-dependent absorption changes at 675 nm of POR-Pchlide-NADPH samples after photoexcitation with a laser pulse centred at ∼475 nm. The average of scans 1–3, scans 8–10 and scans 18–20 are shown to illustrate the formation of the I675* intermediate within approximately 500 ps in the later scans.
(TIF)

Figure S2 Absorption spectra of Pchlide and Chlide. Both Pchlide and Chlide (5 µM) were contained in activity buffer (see Experimental Section).
(TIF)

Figure S3 Pump-probe absorption spectroscopy of POR-Pchlide-NADPH samples after photoexcitation with a laser pulse centred at ∼450 nm. Transient absorption difference spectra at delay times of 4, 19, 489 and 2989 ps after excitation are shown for the average of scans 1–3 (A), scans 8–10 (B) and scans 18–20 (C).
(TIF)

Figure S4 The time-dependent absorption changes at 675 nm of POR-Pchlide-NADPH samples after photoexcitation with a laser pulse centred at ∼450 nm. The average of scans 1–3, scans 8–10 and scans 18–20 are shown to illustrate the lack of I675* formation in approximately 500 ps upon excitation at 450 nm in the later scans.
(TIF)

Figure S5 Absorption spectra and time–dependent absorption changes of enzyme-denatured POR-Pchlide-NADPH samples. (A) Absorption spectra of enzyme-denatured POR-Pchlide-NADPH samples (see Experimental Section) after illumination for varying lengths of time. (B) The time-dependent absorption changes at 675 nm of enzyme-denatured POR-Pchlide-NADPH samples after photoexcitation with a laser pulse centred at ∼475 nm. Samples were illuminated for varying lengths of time prior to denaturation. The data are fitted to 3 exponentials (solid lines).
(TIF)

Figure S6 Pump-probe absorption spectroscopy of Chlide only samples after laser pulse photoexcitation. The laser pulse was centred at ∼450 nm (A) and 460 nm (B). The main panel shows transient absorption difference spectra at delay times of 2, 16, 516 and 2980 ps after excitation. The insets show the respective kinetic transients at 675 nm (black circles) with a fit of the data to a double exponential function (solid line). Time constants of 136 ps (126 ps upon excitation at 460 nm) and 3.5 ns were calculated.
(TIF)

Figure S7 Pump-probe absorption spectroscopy of Pchlide only samples after laser pulse photoexcitation. The laser pulse was centred at ∼450 nm (A), 460 nm (B) and 475 nm (C). Transient absorption difference spectra are shown at delay times of 2, 16, 516 and 2980 ps after excitation.
(TIF)

Figure S8 Pump-probe absorption spectroscopy of samples containing a mixture of 50% Pchlide and 50% Chlide after laser pulse photoexcitation. The laser pulse was centred at ∼435 nm (A), 450 nm (B), 460 nm (C), 475 nm (D) and 580 nm (E). The main panels show transient absorption difference spectra at delay times of 2, 16, 516 and 2980 ps after excitation. The insets show the respective kinetic transients at 675 nm (black circles).
(TIF)

Figure S9 The residuals calculated at all time points after subtraction of the sum of the Pchlide only and Chlide only data from the actual time-resolved difference spectra for all of the Pchlide and Chlide mixtures. Residuals calculated after photoexcitation at 450 nm (A), 460 nm (B) and 475 nm (C). The intensity of the blue region indicates the presence of an additional short-lived negative signal at approximately 675 nm.
(TIF)

Figure S10 Evolution associated difference spectra (EADS) for Pchlide, Chlide and mixtures. EADS resulting from a global analysis of the pump-probe absorption spectroscopy data for Pchlide only, Chlide only, a sum of the ‘Pchlide only’ and ‘Chlide only’ data and a mixture of 50% Pchlide and 50% Chlide after photoexcitation at 450 nm (A), 460 nm (B) and 475 nm (C). The data were fitted to a sequential model which represents the spectral evolution of the decay processes as described in the Materials and Methods Section.
(TIF)

Figure S11 Decay associated difference spectra (DADS) for Pchlide, Chlide and mixtures. DADS resulting from a global analysis of the pump-probe absorption spectroscopy data for Pchlide only, Chlide only, a sum of the ‘Pchlide only’ and ‘Chlide only’ data and a mixture of 50% Pchlide and 50% Chlide after photoexcitation at 450 nm (A), 460 nm (B) and 475 nm (C). The data were fitted to a parallel model of independently decaying components as described in the Materials and Methods Section.
(TIF)

Figure S12 Evolution associated difference spectra (EADS) for POR-Pchlide-NADPH samples. EADS resulting from a global analysis of the pump-probe absorption spectroscopy data for POR-Pchlide-NADPH samples after photoexcitation with a laser pulse centred at 450 nm (A), 460 nm (B) and 475 nm (C). The fits for the average of scans 1–3, scans 8–10 and scans 18–20 are shown. The data were fitted to a sequential model which represents the spectral evolution of the decay processes as described in the Materials and Methods Section.
(TIF)

Figure S13 Decay associated difference spectra (DADS) for POR-Pchlide-NADPH samples. DADS resulting from a global analysis of the pump-probe absorption spectroscopy data for POR-Pchlide-NADPH samples after photoexcitation with a laser pulse centred at 450 nm (A), 460 nm (B) and 475 nm (C). The fits for the average of scans 1–3, scans 8–10 and scans 18–20 are shown. The data were fitted to a parallel model of independently decaying components as described in the Materials and Methods Section.
(TIF)

Figure S14 Fluorescence emission spectra in methanol. Fluorescence emission spectra of samples containing a mixture of 50% Pchlide and 50% Chlide and the sum of the ‘Pchlide only’ and ‘Chlide only’ spectra in methanol after excitation at 460 nm.
(TIF)

Figure S15 Absorption spectra in aqueous buffer. Absorption spectra of samples containing a mixture of 50% Pchlide and 50% Chlide and the sum of the ‘Pchlide only’ and ‘Chlide only’ spectra in aqueous activity buffer.
(TIF)

Figure S16 Fluorescence excitation spectra in aqueous buffer. Fluorescence excitation spectra of samples containing a mixture of 50% Pchlide and 50% Chlide and the sum of the ‘Pchlide only’ and ‘Chlide only’ spectra in aqueous activity buffer using an emission wavelength of 675 nm.
(TIF)
