# Supplementary figures and images for: Mechanistic Reappraisal of Early Stage Photochemistry in the Light-Driven Enzyme Protochlorophyllide Oxidoreductase
Source: PLoS One. 2012 Sep 26;7(9):e45642. doi: 10.1371/journal.pone.0045642 (PMC3458894; doi:10.1371/journal.pone.0045642)

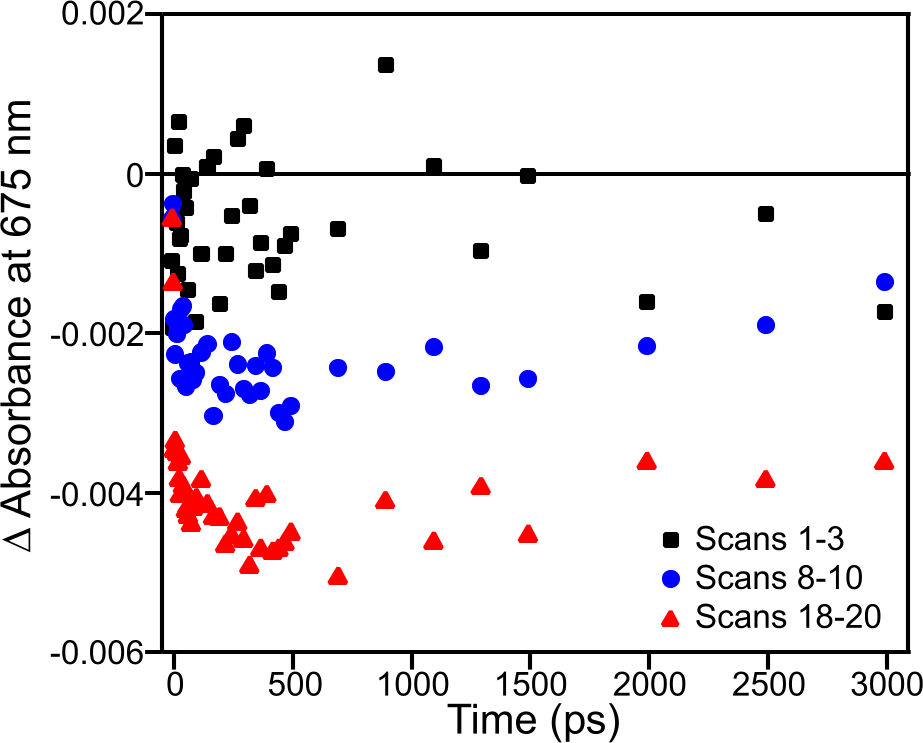

Supplement: Figure S1 — The time-dependent absorption changes at 675 nm of POR-Pchlide-NADPH samples after photoexcitation with a laser pulse centred at ∼475 nm. The average of scans 1–3, scans 8–10 and scans 18–20 are shown to illustrate the formation of the I675* intermediate within approximately 500 ps in the later scans. (TIF) [file pone.0045642.s001.tif]

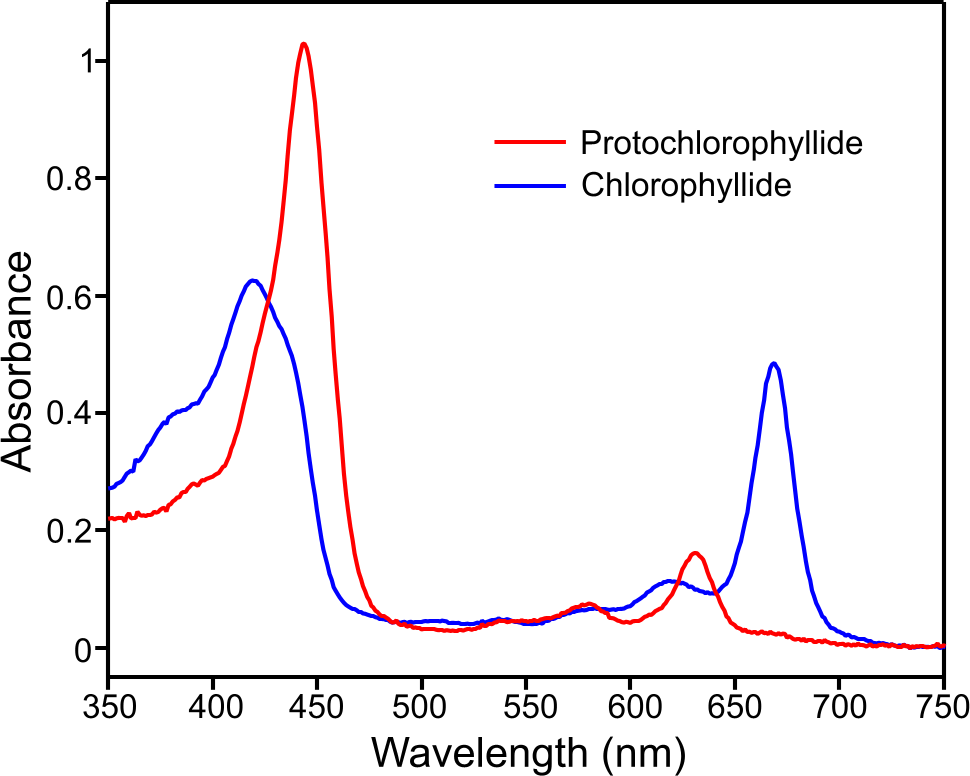

Supplement: Figure S2 — Absorption spectra of Pchlide and Chlide. Both Pchlide and Chlide (5 µM) were contained in activity buffer (see Experimental Section). (TIF) [file pone.0045642.s002.tif]

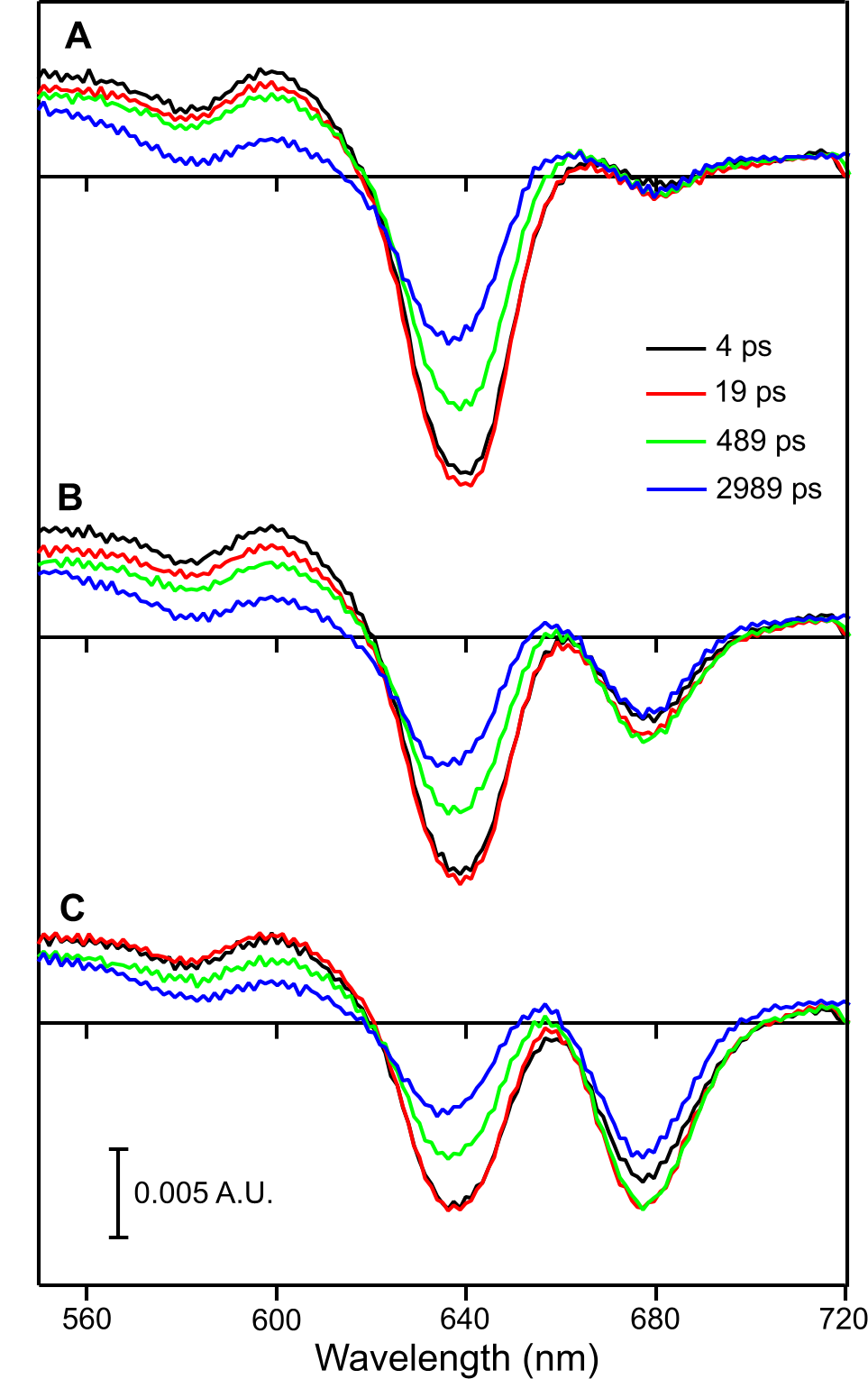

Supplement: Figure S3 — Pump-probe absorption spectroscopy of POR-Pchlide-NADPH samples after photoexcitation with a laser pulse centred at ∼450 nm. Transient absorption difference spectra at delay times of 4, 19, 489 and 2989 ps after excitation are shown for the average of scans 1–3 (A), scans 8–10 (B) and scans 18–20 (C). (TIF) [file pone.0045642.s003.tif]

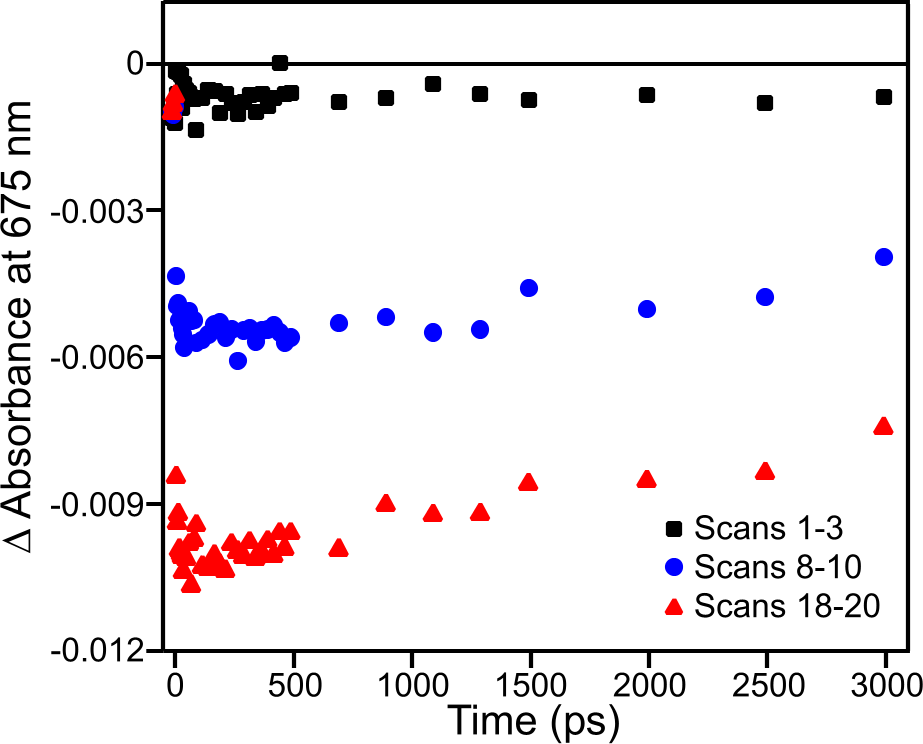

Supplement: Figure S4 — The time-dependent absorption changes at 675 nm of POR-Pchlide-NADPH samples after photoexcitation with a laser pulse centred at ∼450 nm. The average of scans 1–3, scans 8–10 and scans 18–20 are shown to illustrate the lack of I675* formation in approximately 500 ps upon excitation at 450 nm in the later scans. (TIF) [file pone.0045642.s004.tif]

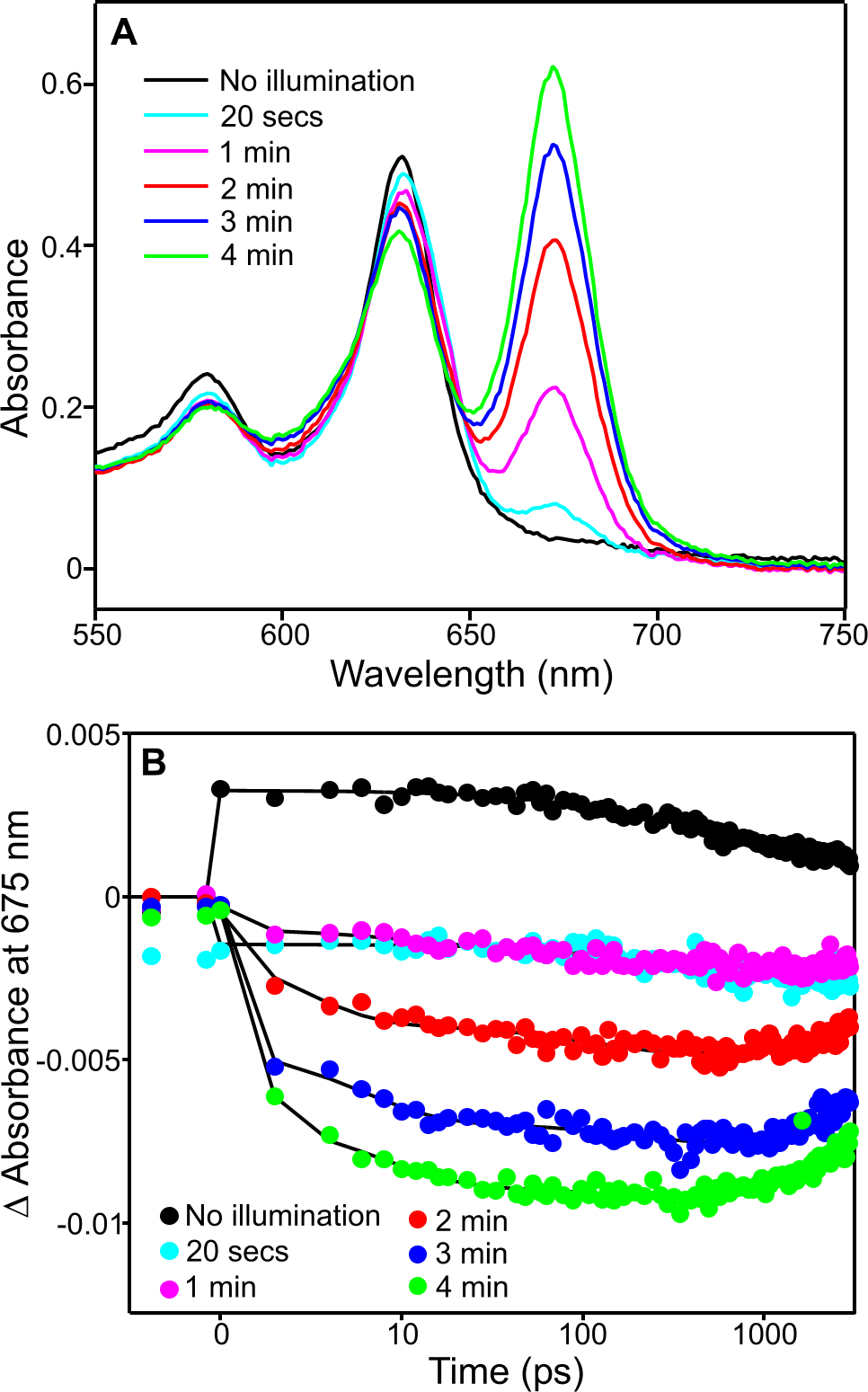

Supplement: Figure S5 — Absorption spectra and time–dependent absorption changes of enzyme-denatured POR-Pchlide-NADPH samples. (A) Absorption spectra of enzyme-denatured POR-Pchlide-NADPH samples (see Experimental Section) after illumination for varying lengths of time. (B) The time-dependent absorption changes at 675 nm of enzyme-denatured POR-Pchlide-NADPH samples after photoexcitation with a laser pulse centred at ∼475 nm. Samples were illuminated for varying lengths of time prior to denaturation. The data are fitted to 3 exponentials (solid lines). (TIF) [file pone.0045642.s005.tif]

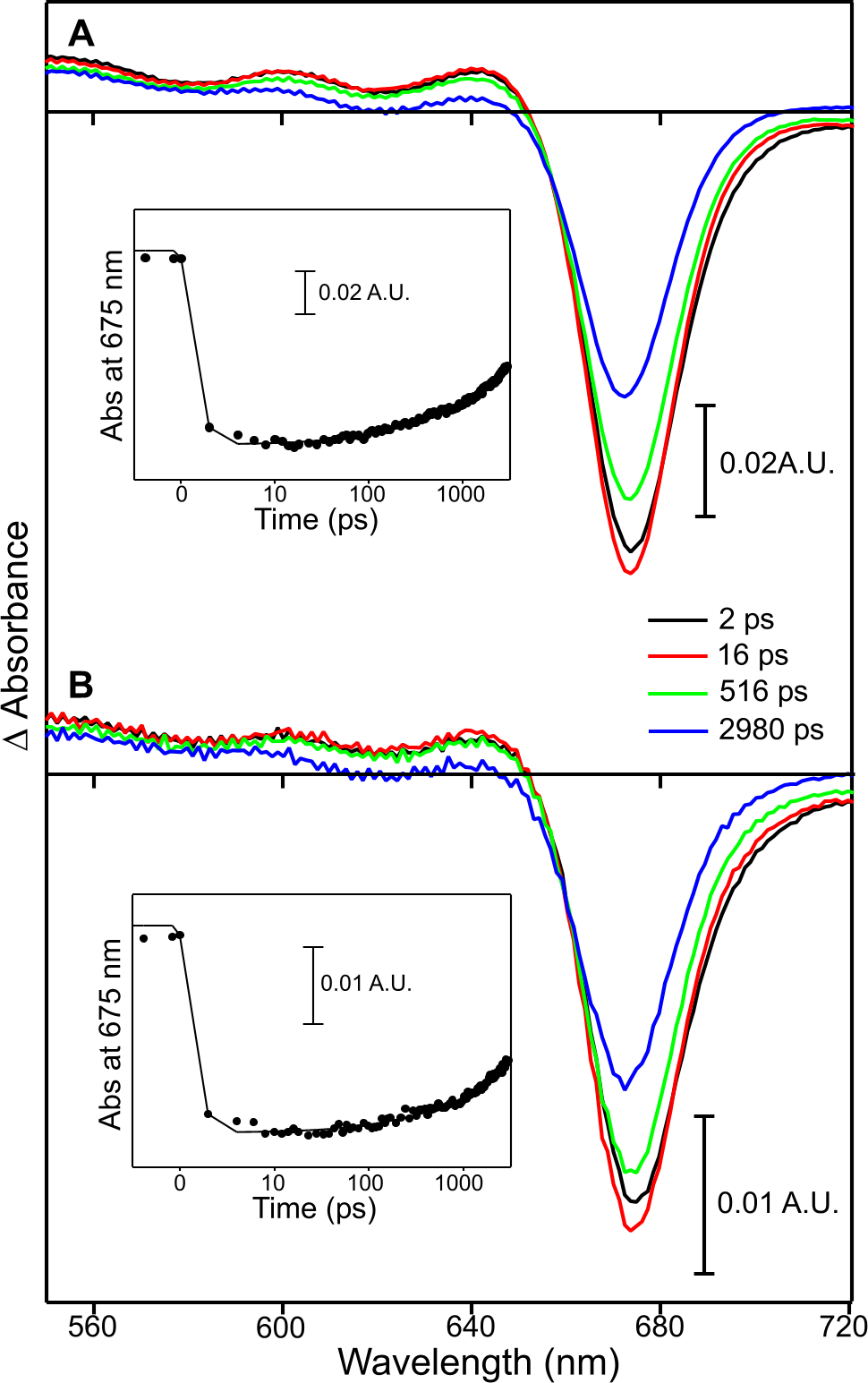

Supplement: Figure S6 — Pump-probe absorption spectroscopy of Chlide only samples after laser pulse photoexcitation. The laser pulse was centred at ∼450 nm (A) and 460 nm (B). The main panel shows transient absorption difference spectra at delay times of 2, 16, 516 and 2980 ps after excitation. The insets show the respective kinetic transients at 675 nm (black circles) with a fit of the data to a double exponential function (solid line). Time constants of 136 ps (126 ps upon excitation at 460 nm) and 3.5 ns were calculated. (TIF) [file pone.0045642.s006.tif]

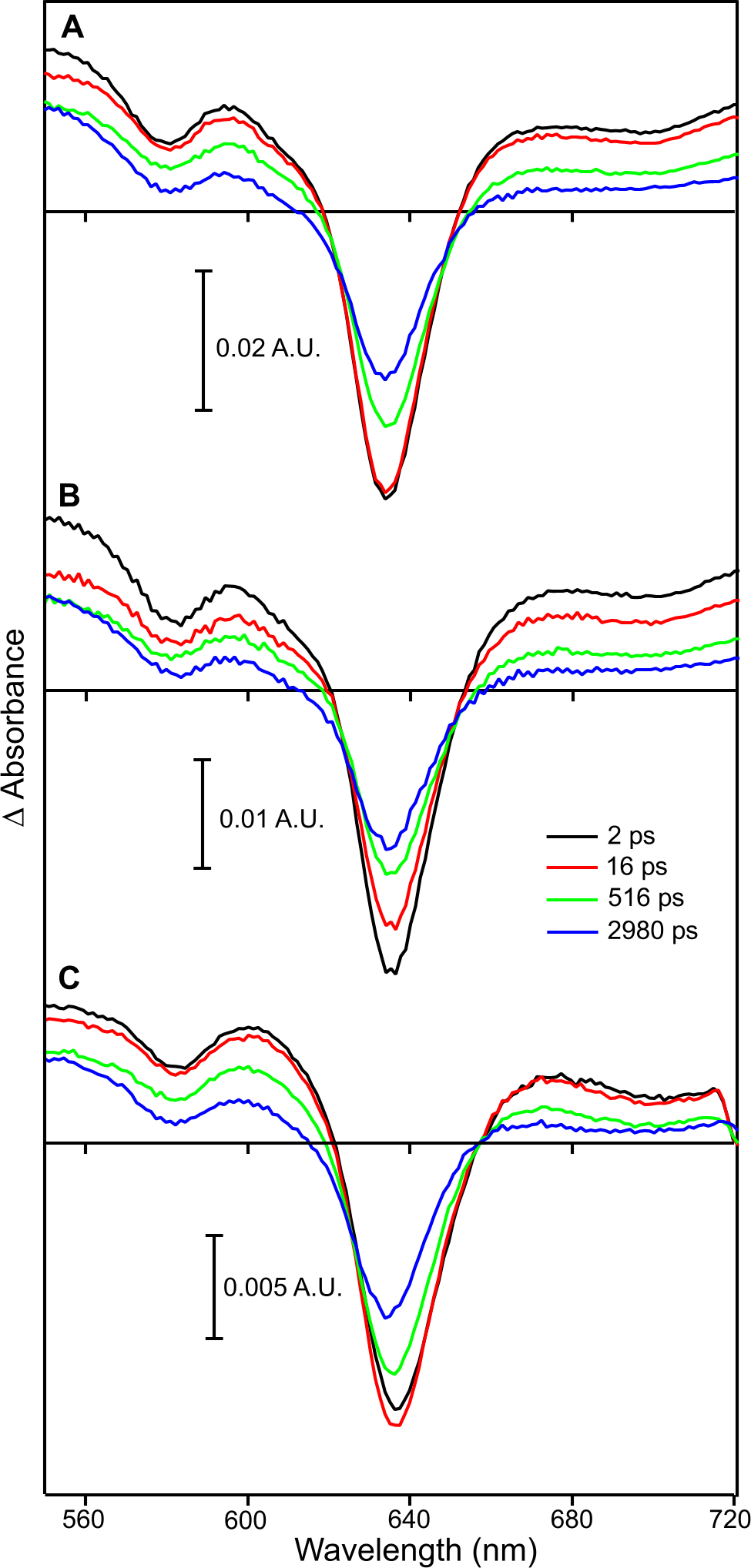

Supplement: Figure S7 — Pump-probe absorption spectroscopy of Pchlide only samples after laser pulse photoexcitation. The laser pulse was centred at ∼450 nm (A), 460 nm (B) and 475 nm (C). Transient absorption difference spectra are shown at delay times of 2, 16, 516 and 2980 ps after excitation. (TIF) [file pone.0045642.s007.tif]

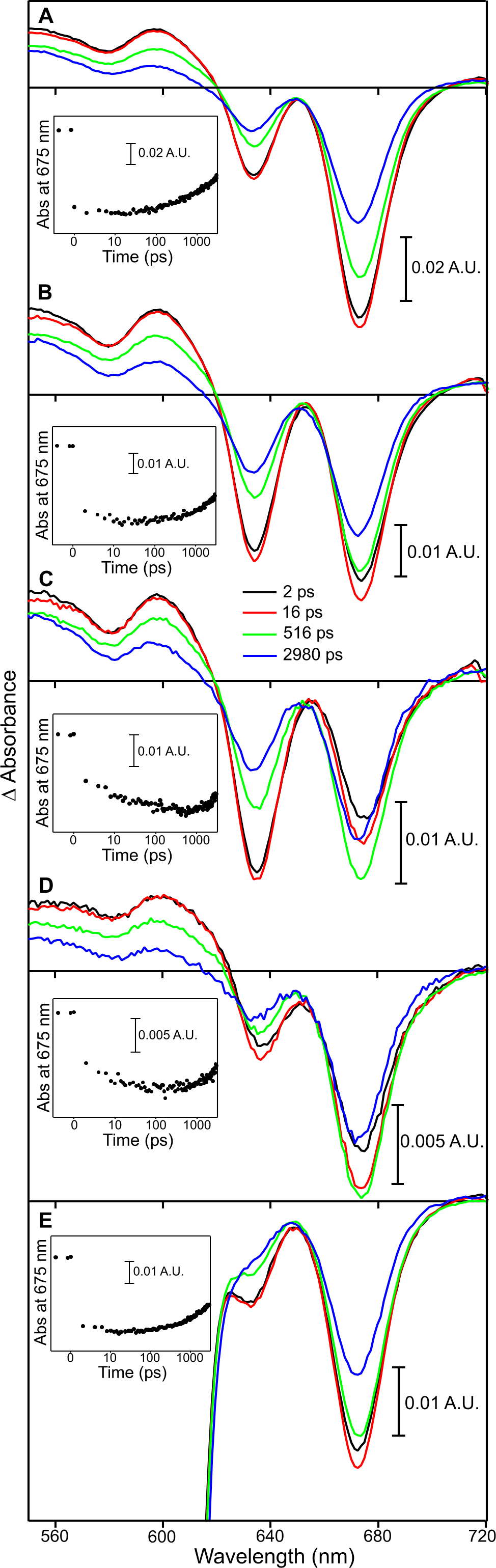

Supplement: Figure S8 — Pump-probe absorption spectroscopy of samples containing a mixture of 50% Pchlide and 50% Chlide after laser pulse photoexcitation. The laser pulse was centred at ∼435 nm (A), 450 nm (B), 460 nm (C), 475 nm (D) and 580 nm (E). The main panels show transient absorption difference spectra at delay times of 2, 16, 516 and 2980 ps after excitation. The insets show the respective kinetic transients at 675 nm (black circles). (TIF) [file pone.0045642.s008.tif]

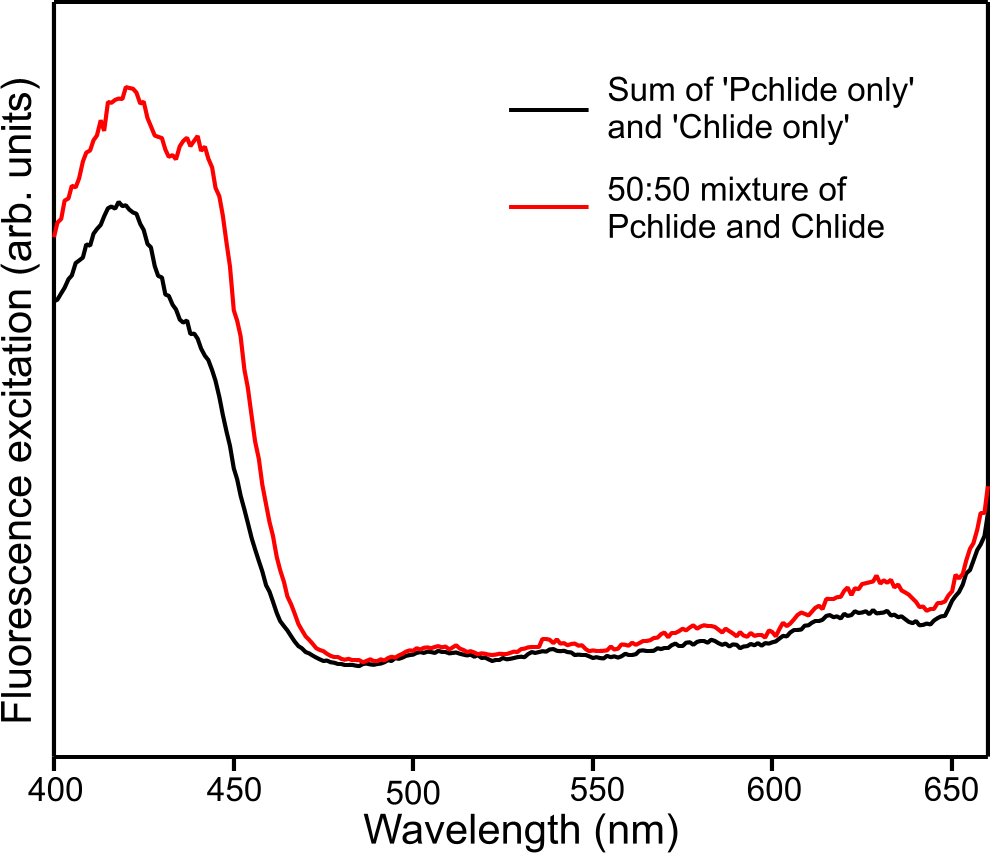

Supplement: Figure S14 — Fluorescence emission spectra in methanol. Fluorescence emission spectra of samples containing a mixture of 50% Pchlide and 50% Chlide and the sum of the ‘Pchlide only’ and ‘Chlide only’ spectra in methanol after excitation at 460 nm. (TIF) [file pone.0045642.s014.tif]

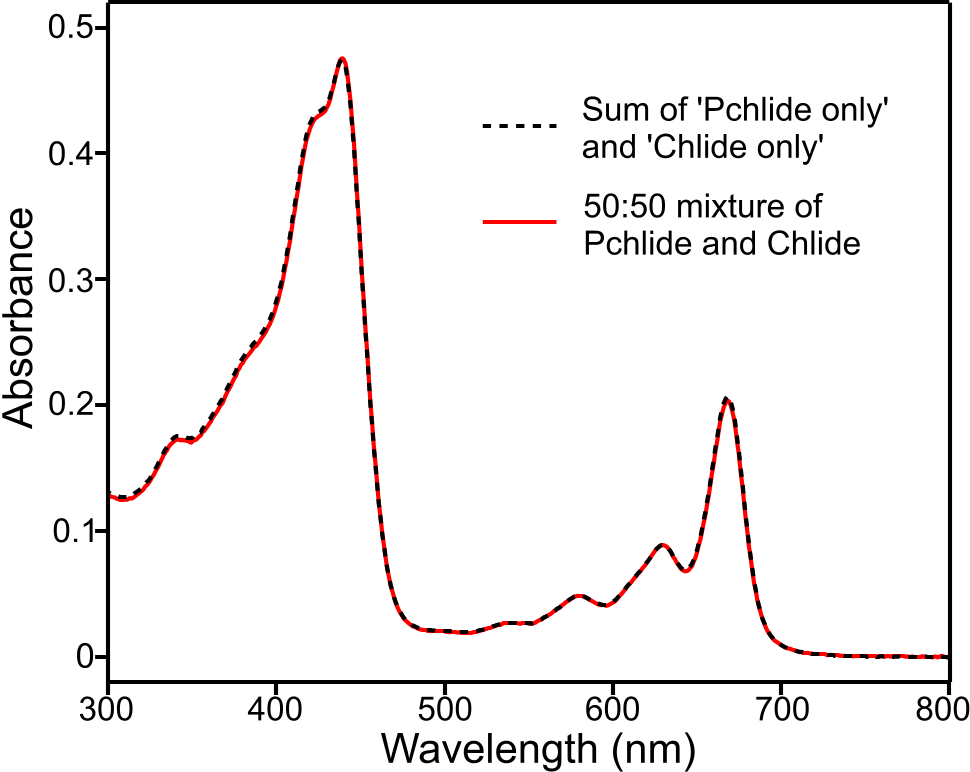

Supplement: Figure S15 — Absorption spectra in aqueous buffer. Absorption spectra of samples containing a mixture of 50% Pchlide and 50% Chlide and the sum of the ‘Pchlide only’ and ‘Chlide only’ spectra in aqueous activity buffer. (TIF) [file pone.0045642.s015.tif]

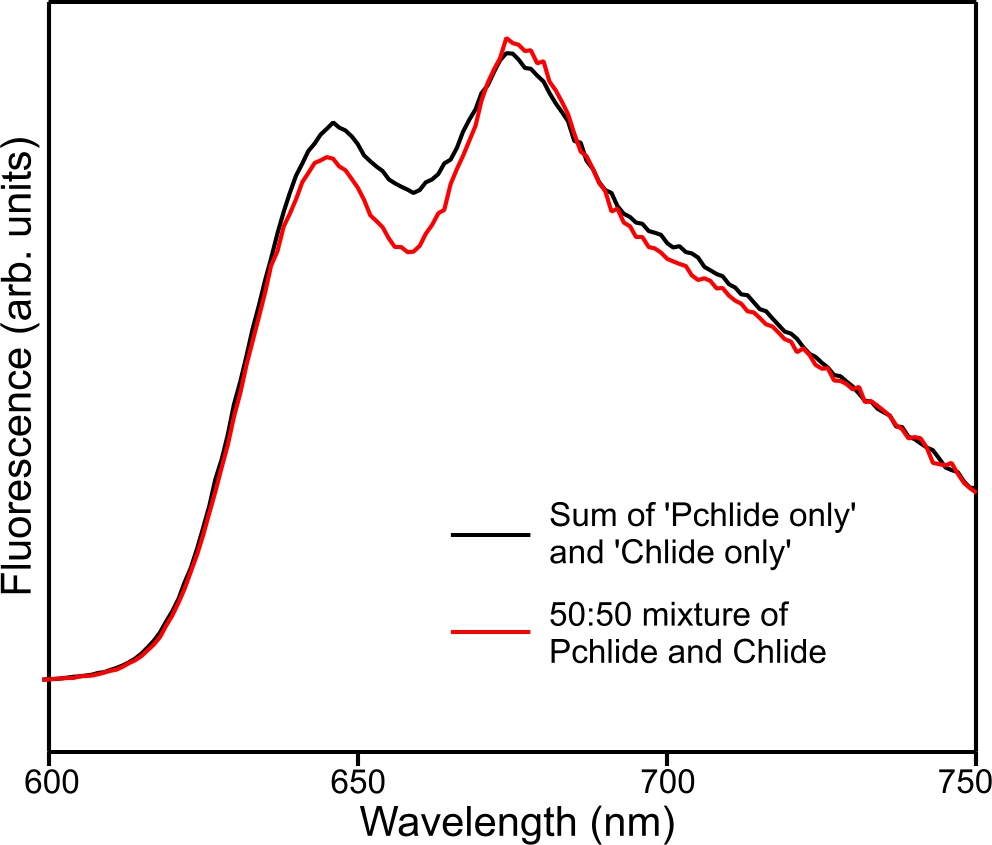

Supplement: Figure S16 — Fluorescence excitation spectra in aqueous buffer. Fluorescence excitation spectra of samples containing a mixture of 50% Pchlide and 50% Chlide and the sum of the ‘Pchlide only’ and ‘Chlide only’ spectra in aqueous activity buffer using an emission wavelength of 675 nm. (TIF) [file pone.0045642.s016.tif]
